# Complete genome sequence of the acetate-degrading sulfate reducer *Desulfobacca acetoxidans* type strain (ASRB2^T^)

**DOI:** 10.4056/sigs.2064705

**Published:** 2011-06-30

**Authors:** Markus Göker, Hazuki Teshima, Alla Lapidus, Matt Nolan, Susan Lucas, Nancy Hammon, Shweta Deshpande, Jan-Fang Cheng, Roxanne Tapia, Cliff Han, Lynne Goodwin, Sam Pitluck, Marcel Huntemann, Konstantinos Liolios, Natalia Ivanova, Ioanna Pagani, Konstantinos Mavromatis, Galina Ovchinikova, Amrita Pati, Amy Chen, Krishna Palaniappan, Miriam Land, Loren Hauser, Evelyne-Marie Brambilla, Manfred Rohde, Stefan Spring, John C. Detter, Tanja Woyke, James Bristow, Jonathan A. Eisen, Victor Markowitz, Philip Hugenholtz, Nikos C. Kyrpides, Hans-Peter Klenk

**Affiliations:** 1 DSMZ - German Collection of Microorganisms and Cell Cultures GmbH, Braunschweig, Germany; 2 DOE Joint Genome Institute, Walnut Creek, California, USA; 3 Los Alamos National Laboratory, Bioscience Division, Los Alamos, New Mexico, USA; 4 Biological Data Management and Technology Center, Lawrence Berkeley National Laboratory, Berkeley, California, USA; 5 Oak Ridge National Laboratory, Oak Ridge, Tennessee, USA; 6 HZI – Helmholtz Centre for Infection Research, Braunschweig, Germany; 7 University of California Davis Genome Center, Davis, California, USA; 8 Australian Centre for Ecogenomics, School of Chemistry and Molecular Biosciences, The University of Queensland, Brisbane, Australia

**Keywords:** anaerobic, mesophile, organoheterotroph, non-motile, sulfate-reducing, sludge bed reactor, *Syntrophaceae*, GEBA

## Abstract

*Desulfobacca acetoxidans* Elferink *et al*. 1999 is the type species of the genus *Desulfobacca*, which belongs to the family *Syntrophaceae* in the class *Deltaproteobacteria*. The species was first observed in a study on the competition of sulfate-reducers and acetoclastic methanogens for acetate in sludge. *D. acetoxidans* is considered to be the most abundant acetate-degrading sulfate reducer in sludge. It is of interest due to its isolated phylogenetic location in the 16S rRNA-based tree of life. This is the second completed genome sequence of a member of the family *Syntrophaceae* to be published and only the third genome sequence from a member of the order *Syntrophobacterales*. The 3,282,536 bp long genome with its 2,969 protein-coding and 54 RNA genes is a part of the *** G****enomic* *** E****ncyclopedia of* *** B****acteria and* *** A****rchaea * project.

## Introduction

Strain ASRB2^T^ (= DSM 11109 = ATCC 700848) is the type strain of the species *Desulfobacca acetoxidans*, which is the type and sole species of its genus *Desulfobacca* [1]. The type strain was isolated from granular sludge of a laboratory-scale upflow anaerobic sludge bed (UASB) reactor fed with acetate and sulfate [1]. The generic name derives from the Neo-Latin word ‘*desulfo*’, meaning desulfuricating, and the Latin word ‘*bacca*’, berry, especially olive, meaning a sulfate-reducing olive-shaped bacterium. The species epithet is derived from the Neo-Latin words ‘*acetum*’, vinegar, and ‘*oxido*’, meaning acetate-oxidizing. The strain is important for the understanding of the competition for acetate between sulfate-reducers and acetoclastic methanogens in sludge [1]. Here we present a summary classification and a set of features for *D. acetoxidans* strain ASRB2^T^, together with the description of the complete genomic sequencing and annotation.

## Classification and features

The single genomic 16S rRNA sequence of *D. acetoxidans* DSM ASRB2^T^ was compared using NCBI BLAST [2,3] under default settings (e.g., considering only the high-scoring segment pairs (HSPs) from the best 250 hits) with the most recent release of the Greengenes database [4] and the relative frequencies of taxa and keywords (reduced to their stem [5]) were determined, weighted by BLAST scores. The most frequently occurring genera were *Desulfobacca* (74.9%) and *Desulfomonile* (25.1%) (4 hits in total). Regarding the two hits to sequences from members of the species, the average identity within HSPs was 98.9%, whereas the average coverage by HSPs was 96.7%. Among all other species, the one yielding the highest score was *Desulfomonile limimaris* (NR_025079), which corresponded to an identity of 90.4% and an HSP coverage of 49.8%. (Note that the Greengenes database uses the INSDC (= EMBL/NCBI/DDBJ) annotation, which is not an authoritative source for nomenclature or classification.) The highest-scoring environmental sequence was AY340836 ('sulfate-reducing fluidized-bed reactor clone SR FBR L13'), which showed an identity of 99.8% and an HSP coverage of 93.0%. The most frequently occurring keywords within the labels of environmental samples which yielded hits were 'sediment' (5.2%), 'microbi' (3.2%), 'lake' (1.9%), 'water' (1.7%) and 'depth' (1.6%) (246 hits in total). The most frequently occurring keywords within the labels of environmental samples which yielded hits of a higher score than the highest scoring species were 'sediment' (5.4%), 'microbi' (2.5%), 'lake' (2.1%), 'water' (1.9%) and 'contamin' (1.8%) (152 hits in total). These keywords reflect some of the ecological and properties reported for strain ASRB2^T^ in the original description [1].

**Figure 1:**
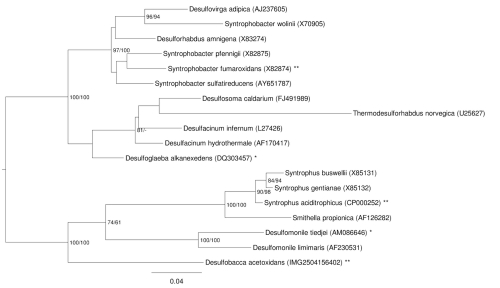
Phylogenetic tree highlighting the position of *D. acetoxidans* relative to the type strains of the other species within the order *Syntrophobacterales*. The tree was inferred from 1,457 aligned characters [6,7] of the 16S rRNA gene sequence under the maximum likelihood (ML) criterion [8]. Rooting was done initially using the midpoint method [9] and then checked for its agreement with the current classification (Table 1). The branches are scaled in terms of the expected number of substitutions per site. Numbers adjacent to the branches are support values from 1,000 ML bootstrap replicates [10] (left) and from 1,000 maximum parsimony bootstrap replicates [11] (right) if larger than 60%. Lineages with type strain genome sequencing projects registered in GOLD [12] are labeled with one asterisk, those also listed as 'Complete and Published' with two asterisks (see [13] and CP000478 for *Syntrophobacter fumaroxidans*).

Figure 1 shows the phylogenetic neighborhood of *D. acetoxidans* in a 16S rRNA based tree. The sequence of the single 16S rRNA gene in the genome differs by 20 nucleotides from the previously published 16S rRNA sequence (AF002671), which contains eleven ambiguous base calls

**Figure 2:**
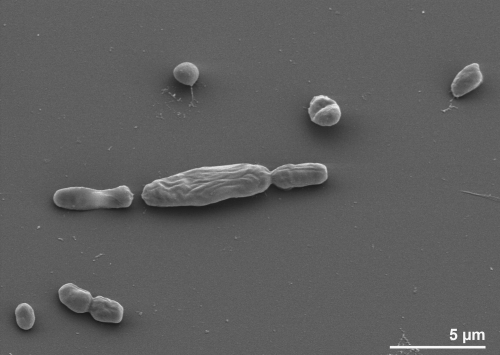
Scanning electron micrograph of *D. acetocidans* ASRB2^T^

Cells of strain ASRB2^T^ are oval to rod-shaped with a size of 1.3 x 1.9-2.2 μm, appear singly or in pairs (Figure 2) and occasionally contain gas vacuoles in the late-exponential growth phase [1]. The strain is non-motile, non-spore-forming and stains Gram-negative (Table 1) [1]. Strain ASRB2^T^ has a temperature range for growth between 27 and 47°C, with an optimum at 36-40°C [1]. At the optimum growth temperature with acetate as sole carbon and energy source the shortest doubling time recorded was 1.7-2.2 days [1]. Growth rate in brackish medium was significantly (4.8 x) slower, and no growth was observed in marine medium [1]. The pH range for growth is 6.5-8.3, with an optimum of pH 7.1-7.5 [1]. Desulfoviridin was not observed, but the c-type cytochromes were present [1]. Sulfate or other inorganic sulfur components serve as electron acceptors *via* reduction to H_2_S [1]. Strain ASRB2^T^ degrades acetate (as the common carbon source and electron donor) completely to CO_2_ *via* the acetyl-CoA/CO-dehydrogenase pathway [1]. The key enzyme of this pathway is encoded by the genes Desac_1965 – Desac_1969. Several more putative electron donors were tested but not found to be utilized by strain ASRB2^T^, such as: propionate, butyrate, lactate, H_2_/CO_2_, formate, ethanol, propanol, butanol, pyruvate, fumarate, glucose, crotonate, benzoate, phenol, aspartate and glutamate [1].

**Table 1:** Classification and general features of *D. acetocidans* ASRB2^T^ according to the MIGS recommendations [14] and the NamesforLife database [15].

MIGS ID	Property	Term	Evidence code
	Current classification	Domain *Bacteria*	TAS [16]
		Phylum *Proteobacteria*	TAS [17-19]
		Class *Deltaproteobacteria*	TAS [20,21]
		Order *Syntrophobacterales*	TAS [21,22]
		Family *Syntrophaceae*	TAS [21,23]
		Genus *Desulfobacca*	TAS [1]
		Species *Desulfobacca acetoxidans*	TAS [1]
		Type strain ASRB2	TAS [1]
	Gram stain	negative	TAS [1]
	Cell shape	oval to rod-shaped	TAS [1]
	Motility	none	TAS [1]
	Sporulation	none	TAS [1]
	Temperature range	27–47°C	TAS [1]
	Optimum temperature	36-40°C	TAS [1]
	Salinity	low salt conditions	TAS [1]
MIGS-22	Oxygen requirement	anaerobic	TAS [1]
	Carbon source	acetate	TAS [1]
	Energy metabolism	organoheterotroph	NAS
MIGS-6	Habitat	fresh water, anaerobic sludge	TAS [1]
MIGS-15	Biotic relationship	free-living	NAS
MIGS-14	Pathogenicity	none	NAS
	Biosafety level	1	TAS [24]
	Isolation	anaerobic granular sludge of a pilot-scale UASB reactor fed with acetate and an excess of sulfate	TAS [1]
MIGS-4	Geographic location	Wageningen, The Netherlands	TAS [1]
MIGS-5	Sample collection time	1995 ore before	NAS
MIGS-4.1	Latitude	51.97	TAS [1]
MIGS-4.2	Longitude	5.67	TAS [1]
MIGS-4.3	Depth	irrelevant	
MIGS-4.4	Altitude	25 m	NAS

Evidence codes - IDA: Inferred from Direct Assay (first time in publication); TAS: Traceable Author Statement (i.e., a direct report exists in the literature); NAS: Non-traceable Author Statement (i.e., not directly observed for the living, isolated sample, but based on a generally accepted property for the species, or anecdotal evidence). These evidence codes are from of the Gene Ontology project [25]. If the evidence code is IDA, the property was directly observed by one of the authors or an expert mentioned in the acknowledgements

### Chemotaxonomy

No data on cell wall structure, quinones, fatty acid pattern or polar lipids are available for this strain.

## Genome sequencing and annotation

### Genome project history

This organism was selected for sequencing on the basis of its phylogenetic position [26], and is part of the *** G****enomic* *** E****ncyclopedia of* *** B****acteria and* *** A****rchaea * project [27]. The genome project is deposited in the Genome On Line Database [12] and the complete genome sequence is deposited in GenBank. Sequencing, finishing and annotation were performed by the DOE Joint Genome Institute (JGI). A summary of the project information is shown in Table 2.

**Table 2:** Genome sequencing project information

**MIGS ID**	**Property**	**Term**
MIGS-31	Finishing quality	Finished
MIGS-28	Libraries used	Four genomic libraries: one 454 pyrosequence standard library, two 454 PE library (8 kb and 12 kb insert size), one Illumina library
MIGS-29	Sequencing platforms	Illumina GAii, 454 GS FLX Titanium
MIGS-31.2	Sequencing coverage	313.2 × Illumina; 37.5 × pyrosequence
MIGS-30	Assemblers	Newbler version 2.3, Velvet 0.7.63, phrap SPS - 4.24
MIGS-32	Gene calling method	Prodigal 1.4, GenePRIMP
	INSDC ID	CP002629
	Genbank Date of Release	April 15, 2011
	GOLD ID	Gc01720
	NCBI project ID	51777
	Database: IMG-GEBA	2504136006
MIGS-13	Source material identifier	DSM 11109
	Project relevance	Tree of Life, GEBA

### Growth conditions and DNA isolation

*D. acetoxidans* ASRB2^T^, DSM 11109, was grown anaerobically in DSMZ medium 728 (*Desulfobacca* medium) [28] at 37°C. DNA was isolated from 0.5-1 g of cell paste using Jetflex Genomic DNA Purification Kit (GENOMED 600100) following the standard protocol as recommended by the manufacturer, but with additional 2 hours incubation with 20 μl proteinase K at 58°C for cell lysis. DNA is available through the DNA Bank Network [29].

### Genome sequencing and assembly

The genome was sequenced using a combination of Illumina and 454 sequencing platforms. All general aspects of library construction and sequencing can be found at the JGI website [30]. Pyrosequencing reads were assembled using the Newbler assembler (Roche). The initial Newbler assembly consisting of 66 contigs in one scaffold was converted into a phrap [31] assembly by making fake reads from the consensus, to collect the read pairs in the 454 paired end library. Illumina GAii sequencing data (1,042 Mb) was assembled with Velvet [32] and the consensus sequences were shredded into 1.5 kb overlapped fake reads and assembled together with the 454 data. The 454 draft assembly was based on 159.0 Mb 454 draft data and all of the 454 paired end data. Newbler parameters are -consed -a 50 -l 350 -g -m -ml 20. The Phred/Phrap/Consed software package [31] was used for sequence assembly and quality assessment in the subsequent finishing process. After the shotgun stage, reads were assembled with parallel phrap (High Performance Software, LLC). Possible mis-assemblies were corrected with gapResolution [30], Dupfinisher [33], or sequencing cloned bridging PCR fragments with subcloning. Gaps between contigs were closed by editing in Consed, by PCR and by Bubble PCR primer walks (J.-F. Chang, unpublished). A total of 55 additional reactions were necessary to close gaps and to raise the quality of the finished sequence. Illumina reads were also used to correct potential base errors and increase consensus quality using a software Polisher developed at JGI [34]. The error rate of the completed genome sequence is less than 1 in 100,000. Together, the combination of the Illumina and 454 sequencing platforms provided 350.7 x coverage of the genome. The final assembly contained 346,781 pyrosequence and 28,710,424 Illumina reads.

### Genome annotation

Genes were identified using Prodigal [35] as part of the Oak Ridge National Laboratory genome annotation pipeline, followed by a round of manual curation using the JGI GenePRIMP pipeline [36]. The predicted CDSs were translated and used to search the National Center for Biotechnology Information (NCBI) non-redundant database, UniProt, TIGRFam, Pfam, PRIAM, KEGG, COG, and InterPro databases. Additional gene prediction analysis and functional annotation was performed within the Integrated Microbial Genomes - Expert Review (IMG-ER) platform [37].

## Genome properties

**Figure 3:**
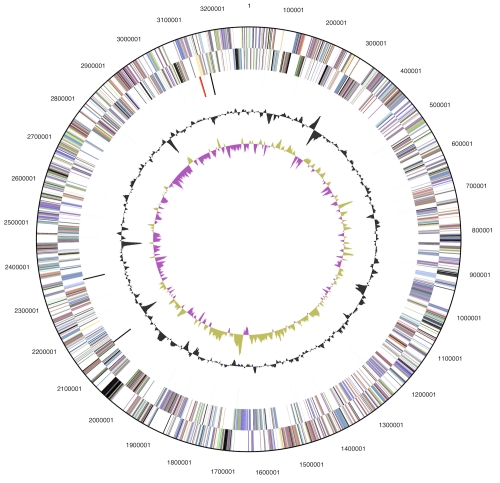
Graphical circular map of the genome. From outside to the center: Genes on forward strand (color by COG categories), Genes on reverse strand (color by COG categories), RNA genes (tRNAs green, rRNAs red, other RNAs black), GC content, GC skew.

The genome consists of a 3,282,536 bp long chromosome with a 52.9% G+C content (Table 3 and Figure 3). Of the 3,023 genes predicted, 2,969 were protein-coding genes, and 54 RNAs; 103 pseudogenes were also identified. The majority of the protein-coding genes (68.2%) were assigned a putative function while the remaining ones were annotated as hypothetical proteins. The distribution of genes into COGs functional categories is presented in Table 4.

**Table 3:** Genome Statistics

**Attribute**	**Value**	**% of Total**
Genome size (bp)	3,282,536	100.00%
DNA coding region (bp)	2,775,726	84.56%
DNA G+C content (bp)	1,736,170	52.89%
Number of replicons	1	
Extrachromosomal elements	0	
Total genes	3,023	100.00%
RNA genes	54	1.79%
rRNA operons	1	
Protein-coding genes	2,969	98.21%
Pseudo genes	103	3.41%
Genes with function prediction	2,063	68.24%
Genes in paralog clusters	507	16.77%
Genes assigned to COGs	2,109	69.77%
Genes assigned Pfam domains	2,213	73.21%
Genes with signal peptides	488	16.14%
Genes with transmembrane helices	726	24.02%
CRISPR repeats	4	

**Table 4:** Number of genes associated with the general COG functional categories

**Code**	**value**	**%age**	**Description**
J	158	7.0	Translation, ribosomal structure and biogenesis
A	1	0.0	RNA processing and modification
K	87	3.9	Transcription
L	136	6.0	Replication, recombination and repair
B	3	0.1	Chromatin structure and dynamics
D	27	1.2	Cell cycle control, cell division, chromosome partitioning
Y	0	0.0	Nuclear structure
V	48	2.1	Defense mechanisms
T	140	6.2	Signal transduction mechanisms
M	200	8.8	Cell wall/membrane/envelope biogenesis
N	14	0.6	Cell motility
Z	0	0.0	Cytoskeleton
W	0	0.0	Extracellular structures
U	82	3.6	Intracellular trafficking and secretion, and vesicular transport
O	92	4.1	Posttranslational modification, protein turnover, chaperones
C	189	8.4	Energy production and conversion
G	89	3.9	Carbohydrate transport and metabolism
E	176	7.8	Amino acid transport and metabolism
F	59	2.6	Nucleotide transport and metabolism
H	135	6.0	Coenzyme transport and metabolism
I	49	2.2	Lipid transport and metabolism
P	116	5.1	Inorganic ion transport and metabolism
Q	32	1.4	Secondary metabolites biosynthesis, transport and catabolism
R	262	11.6	General function prediction only
S	167	7.4	Function unknown
-	914	30.2	Not in COGs
